# Neuronal cell life, death, and axonal degeneration as regulated by the BCL-2 family proteins

**DOI:** 10.1038/s41418-020-00654-2

**Published:** 2020-11-08

**Authors:** James M. Pemberton, Justin P. Pogmore, David W. Andrews

**Affiliations:** 1 Biological Sciences, Sunnybrook Research Institute, Toronto, ON M4N 3M5 Canada; 2 Department of Medical Biophysics, University of Toronto, Toronto, ON M5S 2J7 Canada; 3 Department of Biochemistry, University of Toronto, Toronto, ON M5S 2J7 Canada

**Keywords:** Cell biology, Chemical tools, Neuroscience, Neurological disorders

## Abstract

Axonal degeneration and neuronal cell death are fundamental processes in development and contribute to the pathology of neurological disease in adults. Both processes are regulated by BCL-2 family proteins which orchestrate the permeabilization of the mitochondrial outer membrane (MOM). MOM permeabilization (MOMP) results in the activation of pro-apoptotic molecules that commit neurons to either die or degenerate. With the success of small-molecule inhibitors targeting anti-apoptotic BCL-2 proteins for the treatment of lymphoma, we can now envision the use of inhibitors of apoptosis with exquisite selectivity for BCL-2 family protein regulation of neuronal apoptosis in the treatment of nervous system disease. Critical to this development is deciphering which subset of proteins is required for neuronal apoptosis and axon degeneration, and how these two different outcomes are separately regulated. Moreover, noncanonical BCL-2 family protein functions unrelated to the regulation of MOMP, including impacting necroptosis and other modes of cell death may reveal additional potential targets and/or confounders. This review highlights our current understanding of BCL-2 family mediated neuronal cell death and axon degeneration, while identifying future research questions to be resolved to enable regulating neuronal survival pharmacologically.

## Facts

- Neuronal apoptosis and axon degeneration both occur naturally in development, but also contribute to the pathology of nervous system disease.
- Anti-apoptotic proteins such as BCL-X_L_ and BCL-W can prevent both axon degeneration and apoptosis.
- Many stressors induce neuronal cell death in a PUMA- and BAX-dependent manner.
- Transcriptional upregulation of PUMA is required for axon degeneration.
- BAX, not BAK, is the primary executor of MOMP in neurons.

## Open questions

- Intracellularly, where does MOMP occur after NGF deprivation from axons?
- How do neurons prevent apoptosis while undergoing axon degeneration?
- Why do many stressors trigger neuronal cell death and/or axon degeneration that is PUMA and BAX dependent rather than using other BCL-2 family proteins to?
- Do CNS and PNS axons degenerate through similar mechanisms?
- Can pharmaceutical inhibition of BAX prevent neuronal cell death and improve outcome in neurological events like stroke?
- Does prevention of axon degeneration halt or delay the pathology of neurodegenerative disease?

## Introduction

For many cells, the balance between life and death is regulated by the BCL-2 family of proteins. As reviewed previously, a dance occurs within the BCL-2 family on the mitochondrial outer membrane (MOM) dance floor [1]—with the outcome of this dance ultimately deciding whether a cell will live or die. But what if the dance floor were extended unimaginably? Neurons are morphologically unique cells with long cytoplasmic extensions called axons. The vast distance axons span results in a separation of mitochondrial populations within a single cell—one population within the cell body, and another that extends down the length of the axon. The integrity of both mitochondrial populations is vital to neuronal health [2]. The same BCL-2 family dance-of-death occurs within neurons but now the dance floor has been extended; and as a result, the BCL-2 family can regulate axonal degeneration in addition to life and death. Mitochondria are the “power-house” organelle of the cell, but ironically, the MOM is also the platform to initiate BCL-2-protein regulated cell death [1]. MOM permeabilization (MOMP) results in the release of pro-apoptotic factors into the cytoplasm thereby committing a neuron to die through apoptosis or degenerate only the axon. It remains unclear how the BCL-2 family of proteins regulates this dichotomy of programmed cell death and degeneration, yet it is widespread in the nervous system during development [3] and disease.

## Neuronal cell death and axon degeneration in development

Knockout studies in rodents have solidified the importance of BCL-2 family proteins in the development of the nervous system. Selective deletion of the pro-apoptotic BCL-2 family protein BAX prevents the normal cell death that occurs in the cerebellum and retina, and these mice are reported to have increases in hippocampal and dorsal root ganglion (DRG) neurons [4–7]. Selective deletion of a BAX-activating, pro-apoptotic protein called p53 upregulated modulator of apoptosis (PUMA), also prevents apoptosis of DRG neurons in the peripheral nervous system (PNS). This results in an increased number of neurons and innervating branches during development [8, 9], with no change in expected birth frequency for PUMA knockout mice [10, 11]. Anti-apoptotic proteins of the BCL-2 family also play key roles in development. For example, single or double deletion of the genes encoding proteins BCL-X_L_ and/or MCL-1 results in massive neuronal cell death in the developing central nervous system (CNS) [12–14]. The loss of one allele for *mcl1* plus one allele for *bcl-X* (BCL-X_L_ gene) is sufficient to cause severe brain and craniofacial abnormalities in mouse development suggesting apoptosis must be tightly regulated during development [15]. Moreover, the selective deletion of the BAX-activating protein BIM results in a significantly reduced birth frequency [16]. However, single allele deletion for the *bim* gene rescues the brain and craniofacial abnormalities observed in *mcl1*^+/−^and *bcl*-*x*^+/−^ mice [15], suggesting that BIM may play a role in nervous system development.

BCL-W is an anti-apoptotic protein that contributes to the maintenance of axons [17]. BCL-W knockout mice demonstrate progressive nociceptor sensory neuropathy, and as a result, fail to quickly respond to thermosensation [18]. Intriguing, this neuropathy is due to axon degeneration and occurs without cell body loss, demonstrating a separation between axon degeneration and neuronal cell death [18]. Nevertheless, BCL-W knockout mice are still born at the expected frequency [19], while BCL-X_L_ deficient mice are not viable due to massive apoptosis in the CNS [20]. This suggests BCL-W may play more of a role in pathological settings rather than during development.

Key components of the apoptotic pathway, such as caspases and pro-apoptotic BCL-2 proteins, are downregulated during organismal maturation; rendering most adult tissues, including the brain, resistant to apoptotic stimuli [21]. Additionally, primary cultures of murine hippocampal neurons taken at the embryonic stage develop resistance to apoptotic stimuli as they mature in vitro [21]. Furthermore, we have observed that mature cultures of primary cortical neurons resist death in response to the expression of truncated BIM, a protein with reduced pro-apoptotic activity [22]. However, these cultures of mature neurons remain sensitive to the expression of full-length BIM, demonstrating that mature neurons are resistant, but not entirely refractory to apoptotic stimuli [22]. Resistance to apoptotic stimuli correlating with the maturation of neurons has also been shown in vitro with cultures of sympathetic neurons of the PNS [23–25]. Although mature sympathetic neurons resist apoptosis, they remain permissive to axon degeneration [24] demonstrating that that cell death and axon degeneration can be regulated separately in a single cell. This change in the regulation that occurs during maturation in vitro means that it is important to account for the level of maturity and/or differentiation in experimental design and to report this in experiments using neuron cultures. For instance, stroke mainly occurs in older adults [26], thus it is important for stroke research to be conducted in mature cultures of neurons that have the apoptotic machinery profile more representative of an adult. Additionally, due to the anatomical and biochemical differences between rodent and human neurons [27], mature cultures of human neurons would be the most representative albeit impractical model system. Consequently, there is emerging emphasis on understanding cell death in neurons derived from human stem cell cultures, and grown as brain organoids. Indeed, brain organoids are already proving to be valuable tools for the study of pathology of diseases such as cerebral malaria [28] and microcephaly induced by Zika virus [29].

## Neuronal cell death and axon degeneration in disease

Neuronal cell death and axon degeneration occur naturally during development, however, in the adult they contribute to the pathology of terrible neurodegenerative diseases. In amyotrophic lateral sclerosis (ALS), motor neuron cell death was thought to be the major contributor to disease pathology [8]. However, neuromuscular denervation via axon degeneration prior to cell death has also been shown to be a primary contributor to disease pathology [30]. The genetic mutations that cause ALS prevent proper shuttling of mRNA within axons, disrupting function and leading to cell death [31]. The selective deletion of pro-apoptotic BCL-2 family proteins, such as BAX, BIM, or PUMA, significantly delays disease onset in animal models of ALS [30, 32, 33].

Parkinson’s disease (PD) develops from the specific loss of dopaminergic neurons in the substantia nigra. In pharmaceutically induced models of PD this cell death is regulated by BCL-2 family proteins [34, 35]. Axonal degeneration is also evident in PD and is not just limited to dopaminergic neurons. The early axonal degeneration of serotonergic neurons may contribute to non-motor-related pathologies of PD including anxiety and depression [36].

Apoptosis and axonal degeneration also occur in more acute pathologies such as stroke. Outside of the necrotic core of the infarct in stroke, neurons within the penumbra die from delayed apoptosis, which can be prevented in mouse models by the genetic deletion of BAX [37]. Axonal degeneration has been reported in murine models of stroke [38] and may occur in distinct phases [39].

Wallerian degeneration is a type of axonal degeneration that is thought to occur independent of BCL-2 family proteins [40]. Curiously, Wallerian degeneration can even occur in axons devoid of mitochondria [41] and appears to be independent of BAX and BAK [40]. In response to nerve crush or axotomy, the axon distal to the site of injury undergoes Wallerian degeneration, which involves the activation of calcium-dependent cysteine proteases called calpains that degrade the axon. The portion of the axon proximal to the injury, and still connected to the cell body, remains intact [42]. Here, we mainly discuss BCL-2 family-regulated axon degeneration that requires MOMP and caspase activation (commonly referred to as “pruning”). The contribution of Wallerian degeneration to neurodegenerative disease and injury is discussed elsewhere [43].

## BCL-2 family regulation of MOMP

**Fig. 1:**
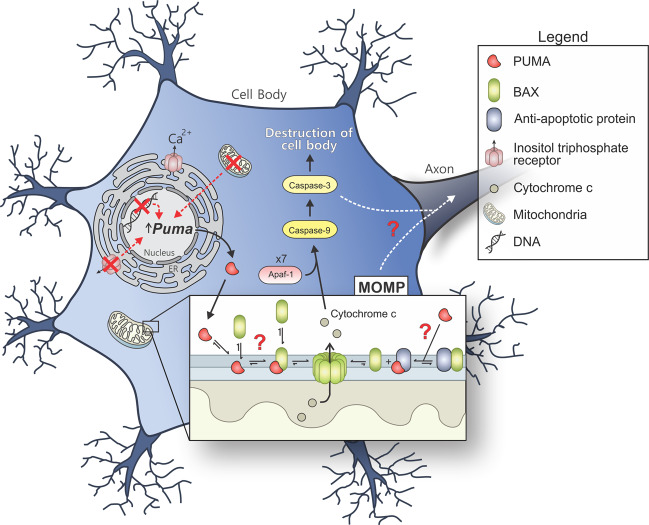
Different stressors/ damage (indicated by a red “X”) induce the transcription and translation of PUMA, resulting in activation of BAX, cytochrome c release and subsequent caspase activation. In neuronal apoptosis it remains uncertain if PUMA activates BAX directly or indirectly. Unknown mechanisms are indicated by a “?”.

MOMP is regulated by interactions among anti-apoptotic (BCL-2, BCL-X_L_, BCL-W, and MCL-1) and pro-apoptotic members of the BCL-2 family proteins. The pro-apoptotic proteins are generally subdivided on the basis of function and the presence of BCL-2 homology (BH) motifs [44] into the pore-formers (BAX and BAK), and the BH3-only proteins (BID, BIM, PUMA, NOXA, BAD, BIK, HRK, etc.). Anti-apoptotic proteins and the pro-apoptotic pore formers contain all four BH motifs (BH1–4), while the BH3-only proteins, as implied from their name, possess only the BH3 motif. Abundance, relative affinity and post-translational modifications all dictate how BCL-2 family proteins interact with each other, ultimately leading to the execution or prevention of MOMP [1]. The embedded together model, reviewed elsewhere [1, 45], posits that these interactions are competitive binding interactions that result in either activation or mutual sequestration mediated inactivation [46–49], the affinities of the requisite interactions are altered by binding to the mitochondrial membrane as the active platform. MOMP occurs upon the activation and oligomerization of the pore-forming proteins BAX and/or BAK on the MOM. BAX and BAK become activated through binding BH3-only “activator” proteins such as BID, BIM, and PUMA. MOMP can be prevented by anti-apoptotic proteins such as BCL-X_L_ and BCL-W by binding to BH3-only activators and/or activated BAX or BAK. As the resulting heteromeric complexes are neither pro nor anti-apoptotic, we refer to this as mutual sequestration [50]. Finally, “sensitizer” BH3-only proteins such as BAD and NOXA promote MOMP by binding to select anti-apoptotic proteins, resulting in the displacement of BH3 activators and active pore formers. Upon BAX activation and subsequent MOMP, cytochrome c is released from the mitochondrial intermembrane space to the cytoplasm where it binds with seven Apaf-1 and caspase-9 molecules in a large complex called an apoptosome. Complex formation activates caspase-9, which, in turn, activates the executioner caspase-3 that degrades many cellular proteins, contributing to apoptosis of the cell (Fig. 1). In addition to cytochrome c, other proteins released into the cytoplasm by MOMP contribute to cell death. Released proteins include apoptosis inducing factor (AIF), endonuclease G (EndoG), SMAC (also called DIABLO), and Omi (also called HtrA2). AIF and EndoG translocate to the nucleus to induce chromatin condensation and DNA fragmentation [51]. SMAC binds to and inhibits x-linked inhibitor of apoptosis protein (XIAP), which normally prevents caspase activity [52]. Omi is a serine protease that cleaves XIAP as well as other target proteins [53].

## PUMA and BAX are the main regulators of neuronal MOMP

The rate limiting step for MOMP is the activation of one or more of the multi-BH motif pore-forming proteins BAX, BAK, or BOK [54]. Surprisingly, in a variety of different neuron cultures the deletion of BAX is sufficient to confer full protection from apoptosis (Table 1), while the deletion of BAK offers no protection [21, 55, 56] and BOK is reported to have no apoptotic role in neocortical neurons [57]. A neuron-specific splice variant of BAK, called N-BAK, has been identified [58], however, the expression of the protein is largely suppressed, and therefore it likely has no apoptotic role [59]. Despite this, in mouse knockout studies the double deletion of both BAX and BAK results in a further accumulation of neurons in the CNS compared to BAX deletion alone [60]. Furthermore, the added deletion of BAK lowers the number of pups that survive into adulthood to <10%. The triple deletion of BAX, BAK, and BOK results in: abnormal brain development and even fewer survivors, but 1% do survive to adulthood [61]. Overall, these knockout studies suggest that BAK and BOK may have a relevant role in the normal apoptosis of the nervous system throughout development. It would be interesting therefore to determine the extent to which BAK and BOK may contribute to axon degeneration rather than playing a major role in survival.

**Table 1:** A variety of stress-types induce PUMA- and BAX-dependent neuronal apoptosis in vitro.

Stress type	Stressor	PUMA and/or BAX dependent	Neuron type	Species	Publication
Oxidative stress	Hydrogen peroxide, 1-methyl-4-phenylpyridinium, TBH, NOC-12	PUMA and BAX	Cortical	Mouse (C57BL/6)	Steckley et al. [35]
	Hydrogen peroxide	PUMA	Cortical	Mouse (C57BL/6)	Baxter et al. [121]
	Hydrogen peroxide	PUMA	Cortical	Mouse (C57BL/6)	Fricker et al. [122]
	6-OHDA	PUMA	Dopaminergic	Mouse (CF-1)	Bernstein et al. [34]
	1-methyl-4-phenylpyridinium	PUMA	Dopaminergic	Mouse (CF-1)	Bernstein and O’Malley [132]
	6-OHDA	BAX	Dopaminergic	Mouse (C57BL/6)	Kim et al. [123]
ER stress	Thapsigargin, tunicamycin	PUMA	Cortical	Mouse (C57BL/6)	Galehedar et al. [65]
	Tunicamycin	PUMA	Telencephalic	Mouse (C57BL/6)	Ghosh et al. [66]
	Tunicamycin	BAX	Superior cervical ganglia	Mouse (C57BL/6)	Smith et al. [124]
	Tunicamycin	PUMA	Cortical	Mouse (C57BL/6)	Concannon et al. [125]
	Thapsigargin, tunicamycin	PUMA	Cortical	Mouse (C57BL/6)	Fricker et al. [122]
	Tunicamycin	PUMA	Motor neuron	Mouse (C57BL/6)	Kieran et al. [32]
DNA damage	Camptothecin	PUMA	Cortical	Mouse (C57BL/6)	Uo et al. [126]
	Camptothecin, etoposide	PUMA	Cortical	Mouse (C57BL/6)	Galehedar et al. [65]
	10 G*y* irradiation	BAX	Cerebral granule neural precursor	Mouse (C57BL/6)	Crowther et al. [56]
	Camptothecin, cisplatin, etoposide	PUMA	Cortical	Mouse (C57BL/6)	Fricker et al. [122]
	Cytosine arabinoside	PUMA and BAX	Superior cervical ganglia	Wistar rat	Wyttenbach et al. [68]
Environmental toxin	Sodium arsenite, okadaic acid	PUMA	Cortical	Mouse (C57BL/6)	Fricker et al. [122]
Proteasomal inhibition	Epoxomicin, bortezomib	PUMA	Cortical	Mouse (C57BL/6)	Tuffy et al. [70]
Stroke-like stress	Oxygen and glucose deprivation	BAX	Cortical	Mouse (C57BL/6)	D’Orsi et al. [37]
Trophic-factor deprivation	Potassium deprivation	PUMA	Cerebellar granule	Mouse (C57BL/6)	Ren et al. [75]
	Potassium deprivation	PUMA	Cerebellar granule	Mouse (C57BL/6)	Ambacher et al. [71]
	Nerve growth factor deprivation	BAX	Superior cervical ganglia	Mouse (C57BL/6)	Deckwerth et al. [7]
Kinase inhibition	Staurosporine	BAX	Superior cervical ganglia	Rat	Deshmukh et al. [127]
	Staurosporine	PUMA	Cortical	Mouse (C57BL/6)	Léveillé et al. [130]
Death receptor	Tumor necrosis factor alpha	PUMA and BAX	Neural precursor cell	Mouse (C57BL/6)	Guadagno et al. [131]

The proteins responsible for BAX activation are the BH3-only “activators” BID, BIM, and PUMA. Despite being a potent direct activator of BAX, there is limited evidence to suggest that BID plays a fundamental role in neuronal cell death [62, 63], and no indication it contributes to axon degeneration [24, 64]. The expression of BIM has been shown to increase in neurons deprived of nerve growth factor (NGF), and in neuronal cultures undergoing ER or oxidative stress [35, 65, 66]. The selective deletion of BIM can delay neuronal apoptosis in some circumstances in vitro, and may play a role in the progression of neurodegenerative disease, as knockout of BIM increases lifespan and delays disease onset in a mouse model of ALS [33]. However, while BIM knockout has been shown to delay neuronal apoptosis upon NGF deprivation, complete protection is afforded by the genetic deletion of BAX [67], suggesting that other factors, in addition to BIM, can result in BAX activation and neuronal apoptosis. Indeed, there is a growing list of publications that demonstrate both neuronal apoptosis and axon degeneration are highly dependent on the BH3-only protein PUMA (Tables 1 and 2). Moreover, numerous publications demonstrate that unlike BIM the single deletion of PUMA prevented both neuronal apoptosis [35, 65, 66, 68–71] and axon degeneration [64] in vitro.

**Table 2:** Axon degeneration requires both PUMA and BAX in vitro.

Stress type	Stressor	PUMA and/or BAX dependent	Neuron type	Species	Publication
Trophic-factor deprivation	NGF deprivation from axons	PUMA	Dorsal root ganglion	Mouse (C57BL/6)	Simon et al. [64]
	NGF deprivation from axons	BAX	Dorsal root ganglion	Mouse (C57BL/6)	Hertz et al. [128]
	NGF deprivation from axons	BAX	Dorsal root ganglion	Mouse (C57BL/6)	Simon et al. [82]
	NGF deprivation from axons	PUMA	Dorsal root ganglion	Mouse (C57BL/6)	Maor-nof et al. [9]
	NGF deprivation from axons	BAX	Dorsal root ganglion	Mouse (C57BL/6)	Nikolaev et al. [129]
	NGF deprivation from axons	BAX	Superior cervical ganglia	Mouse (C57BL/6)	Cusack et al. [24]
BH3-mimetic	ABT-737	BAX	Dorsal root ganglion	Mouse (C57BL/6)	Simon et al. [82]
	ABT-737	PUMA	Dorsal root ganglion	Mouse (C57BL/6)	Simon et al. [64]
	ABT-263	BAX	Dorsal root ganglion	Wistar rat	Cosker et al. [17]

PUMA was first discovered as a potent apoptosis inducing BCL-2 family member transcriptionally regulated by p53 [72, 73] hence its name; the p53 upregulated modulator of apoptosis (PUMA). However, other transcription factors, including CHOP [65] and Foxo3a [35], also regulate the expression of PUMA, enabling its expression in response to multiple varieties of stress in addition to DNA damage [65, 70, 74]. Deficiency of PUMA and/or BAX is sufficient to prevent neuronal cell death in response to oxidative stress, ER stress, DNA damage, environmental toxins, proteasomal inhibition, stroke-like stress, trophic-factor deprivation, pan-kinase inhibition, and death receptor activation (Table 1). PUMA and BAX are also required for axon degeneration induced by local deprivation of NGF (Table 2). The local application of small-molecule inhibitors of anti-apoptotic proteins (termed BH3 mimetics) on axons is sufficient to induce PUMA- and BAX-dependent axon degeneration (Table 2). PUMA and BAX have been demonstrated to be required for apoptosis and axon degeneration in a variety of neuronal cell types from both the CNS and PNS, and across different species (mouse and rat) by multiple independent groups (Tables 1 and 2), suggesting that they play a significant role in regulating neuronal MOMP.

## Regulation of cell death and axon degeneration

**Fig. 2:**
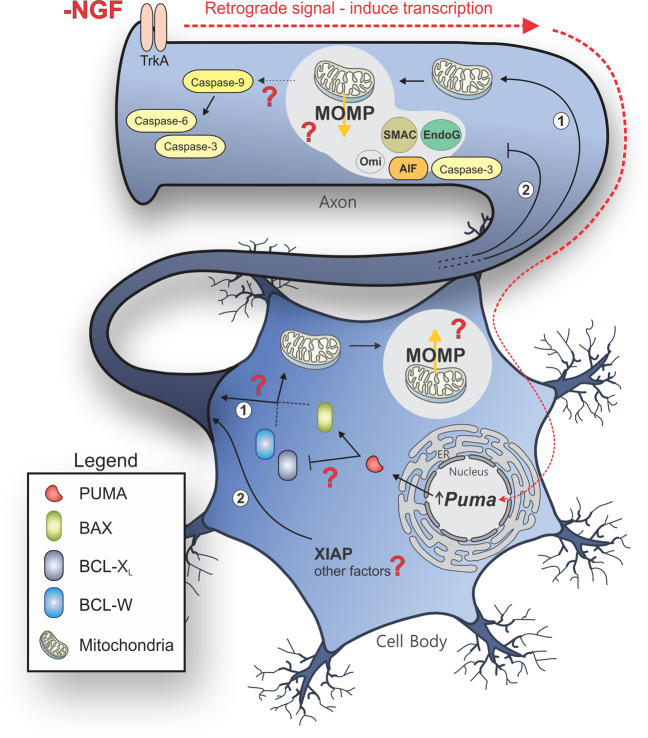
Local deprivation of trophic factor(s) from axons results in a retrograde signal to upregulate transcription of puma. Increased PUMA expression results in either direct or indirect activation of BAX, induction of caspase activation and subsequent degeneration of the axon. However, there are many unknowns in the regulation of selective degradation of axons including: the location of MOMP, how caspase-9 is activated, and how neuronal cell bodies remain alive when PUMA is expressed and caspases activated. Unknown mechanisms are indicated by a “?”. The numbers “1” and “2” indicate the extension of activation and inhibition signals down the axon, respectively.

Often axon degeneration occurs without the death of the neuronal cell body. In order to prevent the neuronal cell death and axon degeneration that contributes to neurodegenerative pathology, it is important to understand what factors determine whether axon degeneration or neuronal apoptosis occurs. One component may be the location of stress. Whole cell trophic-factor deprivation causes both PNS and CNS neurons to die in a PUMA- and BAX-dependent manner [7, 71, 75], however, trophic-factor deprivation exclusively from axons results in degeneration [24]. Using microfluidic chambers that enable separate treatment of neuronal cell bodies and axons, trophic-factor deprivation from axons results in transcriptional upregulation of key genes required for axon degeneration such as *bbc3*, encoding PUMA [9, 64] (Fig. 2). PUMA expression de novo in the cell body is required for axon degeneration as application of the transcriptional inhibitor Actinomycin D prevents trophic-factor withdrawal-induced axon degeneration [9, 64]. PUMA is a BH3-only protein and, thus, is predicted to function as either a direct activator or a sensitizer (indirect activator) of BAX and BAK. Numerous publications have shown that the BH3-domain of PUMA can directly bind to and activate BAX, resulting in MOMP [75–77]. In addition, PUMA can also bind to and inhibit all anti-apoptotic proteins [78]. Therefore, PUMA has two apoptotic functions to execute MOMP (activator and sensitizer), but which of these functions occur during neuronal apoptosis or axon degeneration? The reported role for PUMA in apoptosis and axon degeneration may be related to the location of BCL-2 family proteins. Biochemical fractionation has determined that the anti-apoptotic proteins BCL-2 and MCL-1 are primarily localized within the cell body of cultured murine DRG neurons, while BCL-X_L_ and BCL-W can be found in both the cell body and axon [18, 64]. Localization of mitochondria within the axon may also play a relevant role. For example, in PNS neurons, accumulations of mitochondria are consistently observed at nodal junctions between myelin on the axon and at the synapse, both areas that are rich in membrane channel proteins and participate in the generation of action potentials [79]. However, CNS neurons appear to have more axonal mitochondria at intermodal areas [80]. Could MOMP selectively occur to one cluster of mitochondria within the axon, and not perpetuate down the length of the axon? Or does an apoptotic trigger wave occur spreading down the entire length of the axon [81]? It makes intuitive sense that MOMP occurring in the cell body would induce apoptosis, while selective MOMP in the axon could result in axon degeneration (Fig. 2). Indeed, selective application of BH3-mimetic to axons results in caspase activation and subsequent degeneration [17, 64, 82]; suggesting that axonal MOMP is sufficient to induce degeneration. Additionally, because BH3 mimetics work by inhibiting anti-apoptotic proteins, these data suggest that the sensitizer function of PUMA would be sufficient to induce axon degeneration. Immunofluorescence assays have also shown that trophic-factor deprivation from axons results in cytochrome c release [24], but whether MOMP is exclusive to the axon remains to be determined. Western blot and mass spec demonstrate that PUMA (protein) can be detected in the cell body while evidence for synthesis in or localization to the axon is less clear [9, 64]. PUMA is required for CNS neurons to die from trophic-factor deprivation (Table 1), however, a role for PUMA in CNS neuron axon degeneration has not been determined. In PNS neurons, selective application of the translational inhibitor cycloheximide to the cell body prevents axon degeneration, thus de novo PUMA expression in the cell body is required for axon degeneration [64]. However, it is unknown how the expression of PUMA in the cell body of PNS neurons results in axonal MOMP, while sparing the cell body (Fig. 2). Furthermore, neurons somehow restrict the spillover of active caspases from the axon into the cell body. A role has been speculated for the endogenous caspase inhibitor XIAP in preventing active caspases reaching the cell body [24], but how XIAP is regulated in apoptosis versus axon degeneration remains unclear. Additionally, it is unclear how neuronal cell death is prevented in the cell body subsequent to MOMP mediated release of other mitochondrial proteins including many implicated in cell death-including cytochrome c, EndoG, Smac, and Omi (Fig. 2). One key difference between the CNS and PNS is the remarkable ability of PNS neurons to regenerate after xonal degeneration [83]. In the PNS, regeneration of the axon is possible as long as the neuronal cell body remains alive. Thus, future research should investigate whether in CNS neurons the cell bodies die after axon degeneration, potentially explaining one reason why regeneration in the CNS is so poor. If so then understanding of how PNS neuronal cell bodies survive becomes even more important.

Another relevant factor may be assembly of the apoptosome at the onset of neuronal apoptosis [24, 64] (Fig. 1), but not axonal degeneration [24] (Fig. 2). Mature neurons restrict the expression of Apaf-1 through chromatin remodeling limiting caspase-9 activation by MOMP [84]. However, during neuronal degeneration, activation of caspase-9 occurs by an as yet unknown mechanism [24, 82]. The activation of executioner caspases and subsequent apoptosis has been reported to occur in other cell types genetically deficient in either Apaf-1 or caspase-9 [85].

## Pharmacological inhibition of apoptotic machinery—implications for neurodegenerative disease

Activation of BAX is required in both axon degeneration and neuronal cell death. However, attempts at therapies preventing cell death in response to neurological damage have targeted steps either upstream or downstream of the coordination of BCL-2 family proteins at the mitochondria. For example, Foretinib, a pan-kinase inhibitor, was identified in a screen for kinase inhibitors that could reduce cell death and axon degeneration in rat superior cervical ganglion cultures [86]. Foretinib inhibits both trophic-factor deprivation-induced axon degeneration and Wallerian degeneration (due to axotomy). However, Foretinib delayed but did not prevent axon degeneration in vivo upon sciatic nerve-crush experiments in rats [86].

In contrast, in ischemia and reperfusion injury, the inhibition of JNK signaling has been an attractive target to prevent apoptosis for the protection of tissues [87, 88]. Inhibiting JNK3 has been proposed for treating ALS as JNK inhibition prevents the apoptosis of motor neurons derived from human iPS cells [89]. Consistent with potential utility in ALS, the selective JNK inhibitor SP600125 protects CNS neurons from axon degeneration induced by trophic-factor withdrawal [90]. Although cardioprotective and neuroprotective activities have been observed for synthetic small-molecule inhibitors of JNKs [87], systemic administration of current JNK inhibitors is expected to suffer from on-target side effects because different members of the JNK family exert diverse physiological properties. Therefore, to be useful as neuroprotectants it will be necessary to make inhibitors with exquisite isoform specificity. Recent structure activity relationship studies resulted in an inhibitor that is somewhat more specific for JNK3 suggesting selectivity may be achievable [91].

**Fig. 3:**
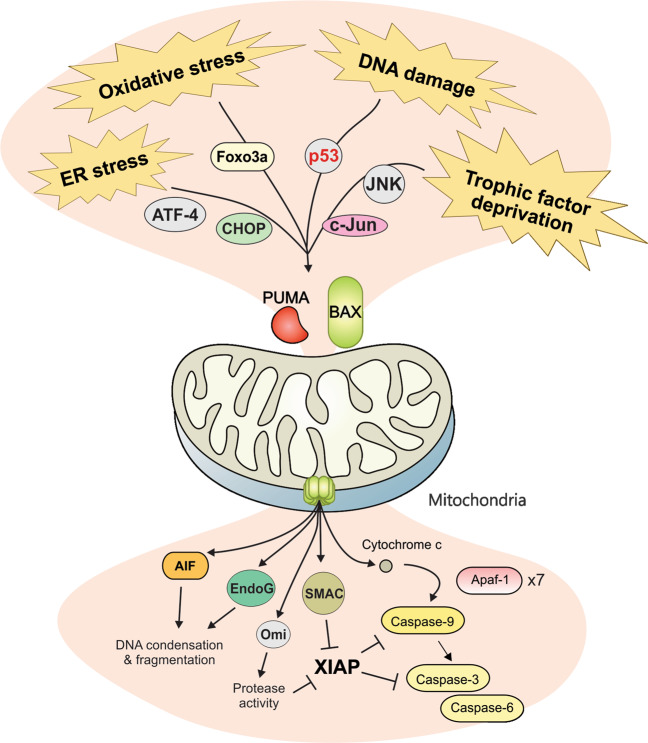
Rationale for the development of BAX inhibitors. Upstream signaling pathways converge at the MOM with BAX activation as the commitment step in apoptosis. Preventing caspase activation is not sufficient as MOMP causes the release of pro-apoptotic proteins that can lead to caspase independent cell death.

In contrast to these inhibitors that act upstream of MOMP, attempts to inhibit the function of caspases downstream of MOMP met limited success. Treatment of NGF deprived murine neurons with pan-caspase inhibitor Z-VAD-FMK only partially protected neurons from cell death or axonal degradation compared to BAX knockout neurons [92]. This result is consistent with the release of multiple pro-apoptotic molecules from mitochondria by MOMP (Fig. 3) and suggests that the prevention of MOMP would be a more effective strategy than inhibiting apoptosis downstream of MOMP [93]. Thus, pharmacologic targeting of BAX may be an efficient way to limit cell death, as BAX activation is required for MOMP induced apoptosis (Fig. 3).

Until recently only the inhibitory BCL-2 family proteins have been selectively targeted by compounds. The success of the BCL-2 inhibitor Venetoclax, in patients with chronic lymphocytic leukemia, small lymphocytic lymphoma, and as a combinatorial therapy in acute myeloid leukemia [94–96], clearly demonstrates that the protein–protein interactions of BCL-2 family proteins are druggable. Venetoclax and other inhibitors of anti-apoptotic proteins are referred to as BH3 mimetics because they were designed to mimic the BH3 motif of BAD, and thereby function as competitive inhibitors for binding of BH3 proteins and active BAX and BAK to anti-apoptotic proteins [97]. As activation of BAX is rate limiting for MOMP and is regulated by multiple BCL-2 family members upstream of oligomerization, Bax is an ideal post-insult target (Tables 1 and 2). Genetic deletion of BAX does not prevent cell injury but has been shown to prevent neuronal cell death in mouse models of stroke and traumatic brain injury [37, 98]. Thus, small peptides based on BH3 motifs that inhibit rather than activate BAX, similar to those identified for BAK [99], or synthetic small molecules that bind interfaces on BAX allosteric to the canonical BH3-groove interface would be very useful tools. The small-molecule inhibitor BAI1 binds at a novel, allosteric pocket between the α5–α6 hairpin and the loop between α3 and α4 [100]. Microscale thermophoresis measurements and chemical shift perturbations in ^15^N-^1^H HSQC nuclear magnetic resonance experiments demonstrate that BAI1 binds BAX directly. Although BAI1 appears to inhibit BAX and not BAK, given the similar fold between BCL-2 family members it remains to be formally determined whether BAI1 will have off-target effects due to binding of other BCL-2 family proteins [101]. The in vivo activity of BAI1 to prevent apoptosis is limited. Nonetheless, in zebrafish and mice, BAI1 protects against doxorubicin-induced cardiomyopathy as measured by a reduction in caspase-3 cleavage and retention of mitochondrial polarization [102]. The effects of BAI1 in cerebral injury or ischemia-reperfusion models have yet to be demonstrated but provide an exciting opportunity that may drive further development of this early stage synthetic.

Inhibition of BAX prior to activating conformation changes such as oligomerization and insertion into membranes is attractive because in neurons it would prevent all of the pro-apoptotic BH3 proteins from triggering MOMP and therefore also prevent release of other pro-apoptotic proteins from mitochondria (Fig. 3). In addition, BAX activation can occur independent of BH3-only proteins by heat or a variety of small molecules suggesting there may be as yet undiscovered effectors of BAX in cells [103, 104]. BAX and BAK execution of MOMP has also been demonstrated in cultured cells in which the genes encoding all known BH3-only proteins have been deleted [105], further stressing the importance of targeting the executioner proteins directly as opposed to other upstream signaling proteins.

Currently, there is only one study demonstrating that it is possible to inhibit both BAX and BAK with a single compound. MSN-50 and MSN-125 were identified as BAX oligomerization inhibitors and shown to prevent cell death of cortical neurons from glutamate excitotoxicity [106]. Inhibition of BAX and BAK with these molecules protected cells long enough that they were able to recover from an otherwise lethal exposure to Actinomycin D or Staurosporine.

BAI1 and the oligomerization inhibitors MSN-50 and MSN-125 are early stage tool compounds with micromolar affinities- with off-target effects at effective concentrations. Nonetheless, it may be possible to use BAI1 to probe the role of BAX in axonal degeneration. Most important, BAI1 enabled identification of the binding pocket on BAX which will likely lead to optimization of these inhibitors, or new molecules.

Given the emergence of these inhibitors, the question remains if pharmacological BAX inhibition protects against neurological events such as stroke. After the excitotoxic cell death characteristic of ischemic injury from a stroke, there is a transitional period where a reduction of local cerebral blood flow results in programmed cell death over a number of days, to weeks [107–109]. This time period provides a unique opportunity for a BAX inhibitor to limit the amount of cell death following stroke. However, it remains unknown whether pharmaceutical inhibition of BAX can prevent axonal degeneration, and if preventing this axonal degeneration will translate to better outcome for patients. If BAX inhibition is demonstrated to provide therapeutic benefit it would provide the impetus for optimization of specificity, affinity, and pharmacological properties of a BAX inhibitor.

The initial reaction to inhibiting BAX and BAK as a treatment for neurological diseases of all kinds including neurodegenerative diseases such as ALS has been skepticism as BAX and BAK are predicted to be tumor suppressors. However, BAX deficiency alone does not result in an increased risk to spontaneous cancer formation in mice [110]. Additionally, an acute insult such as ischemia or stroke would only require transient BAX inhibition, further limiting risk to cancer development. Given the exquisite dependence of neuronal cell death on PUMA and BAX an attractive approach would be to target this protein pair specifically, which may limit undesirable on-target effects in other tissues. Only with the development of better tool compounds will it be possible to address both the potential benefits and issues related to inhibiting neuronal apoptosis.

## Noncanonical roles of BCL-2 family proteins in neurons

BCL-2 family proteins are well known for their role in regulating MOMP, resulting in caspase activation and committing a neuron to either apoptosis or axon degeneration. However, other types of cell death exist which proceed independently of caspase activation [111, 112], but may still be regulated in part by noncanonical functions of BCL-2 family proteins. Indeed, other types of cell death such as necroptosis and ferroptosis contribute to the pathology of neurological disease and trauma, such as hemorrhagic stroke [113]. Necroptosis signaling has been shown to contribute to the progression of Wallerian degeneration of both CNS and PNS neurons [114], a process that when induced through nerve-crush experiments, also results in a transcriptional upregulation of PUMA [9]. In colorectal cancer cell lines PUMA can enhance necroptosis signaling by inducing the release of mitochondrial DNA to the cytoplasm where it is recognized by DNA sensors DAI/Zbp1 and STING, leading to enhanced signaling by RIP3 and phosphorylation of MLKL [115]. Curiously, genetic deletion of BAX and BAK had no effect on inhibiting this necroptosis, suggesting that PUMA acts in a noncanonical fashion to induce necroptosis [115]. This is in stark contrast with another study showing that the genetic deletion of BAX is sufficient to prevent necroptosis in mouse embryonic fibroblasts [116]. Further research is required to determine whether PUMA and/or BAX contribute to necroptosis signaling in neuronal cell death and/or axon degeneration (Wallerian), and whether pharmacological inhibition of either protein can prevent non-apoptotic neuron death. If so, inhibition of BAX may be sufficient to prevent multiple types of neuronal cell death, and multiple types of axon degeneration. It is also vital to understand the mechanism by which BAX is required for necroptosis, as current small-molecule inhibitors such as MSN-125 prevent BAX oligomerization. If BAX oligomerization is not required for necroptosis, an alternative strategy will be needed to inhibit this form of cell death. Ferroptosis is a MOMP independent, iron-dependent form of programmed cell death that occurs upon the accumulation of lipid peroxidation [117]. Conditional deletion of the antioxidant enzyme glutathione peroxidase 4 results in rapid motor neuron cell death through ferroptosis and paralyzes mice, suggesting that the inhibition of ferroptosis may play a role in the response to oxidative stress in adult motor neurons in vivo [118]. Moreover, ferroptosis kills a large percentage of the cells during transdifferentiation of somatic cells, such as fibroblasts, into neurons [119]. Surprisingly, overexpression of either BCL-2 or BCL-X_L_ improved the efficiency of neuron conversion by reducing the levels of reactive oxygen species (ROS), and preventing ferroptosis [119]. How anti-apoptotic proteins reduce ROS is unknown, however, the expression of BCL-2 mutants such as one in which serine 70 was replaced with alanine exhibited reduced BAX binding but increased activity at preventing ferroptosis, suggesting that BCl-2 and BCL-X_L_ prevent ROS accumulation through a noncanonical role [119].

Chemotherapy-induced peripheral neuropathy is a form of pathological axon degeneration that occurs in patients treated with the chemotherapeutic paclitaxel. Paclitaxel treatment of cultured sensory neurons prevents the axonal transport of *bclw* mRNA, reducing protein levels and resulting in peripheral neuropathy [120]. BAX may also be involved as knockdown significantly reduced axon degeneration induced by paclitaxel. Interestingly, loss of axonal BCL-W was reported to result in aberrant calcium signaling, possibly from the IP_3_ receptor at the ER, and subsequently calpain activation [120]. Consistent with this, release of cytochrome c from axonal mitochondria was not observed in cultured neurons treated with paclitaxel [120]. Together, these data suggest that BCL-W and BAX act independently of MOMP to regulate peripheral neuropathy. Thus, caspase inhibitors are unlikely to be useful as MOMP and subsequent caspase activation do not contribute to this form of axon degeneration. However, a BAX inhibitor may be of clinical use because the knockdown of BAX protected cultured axons from neuropathy.

## Conclusion

**Fig. 4:**
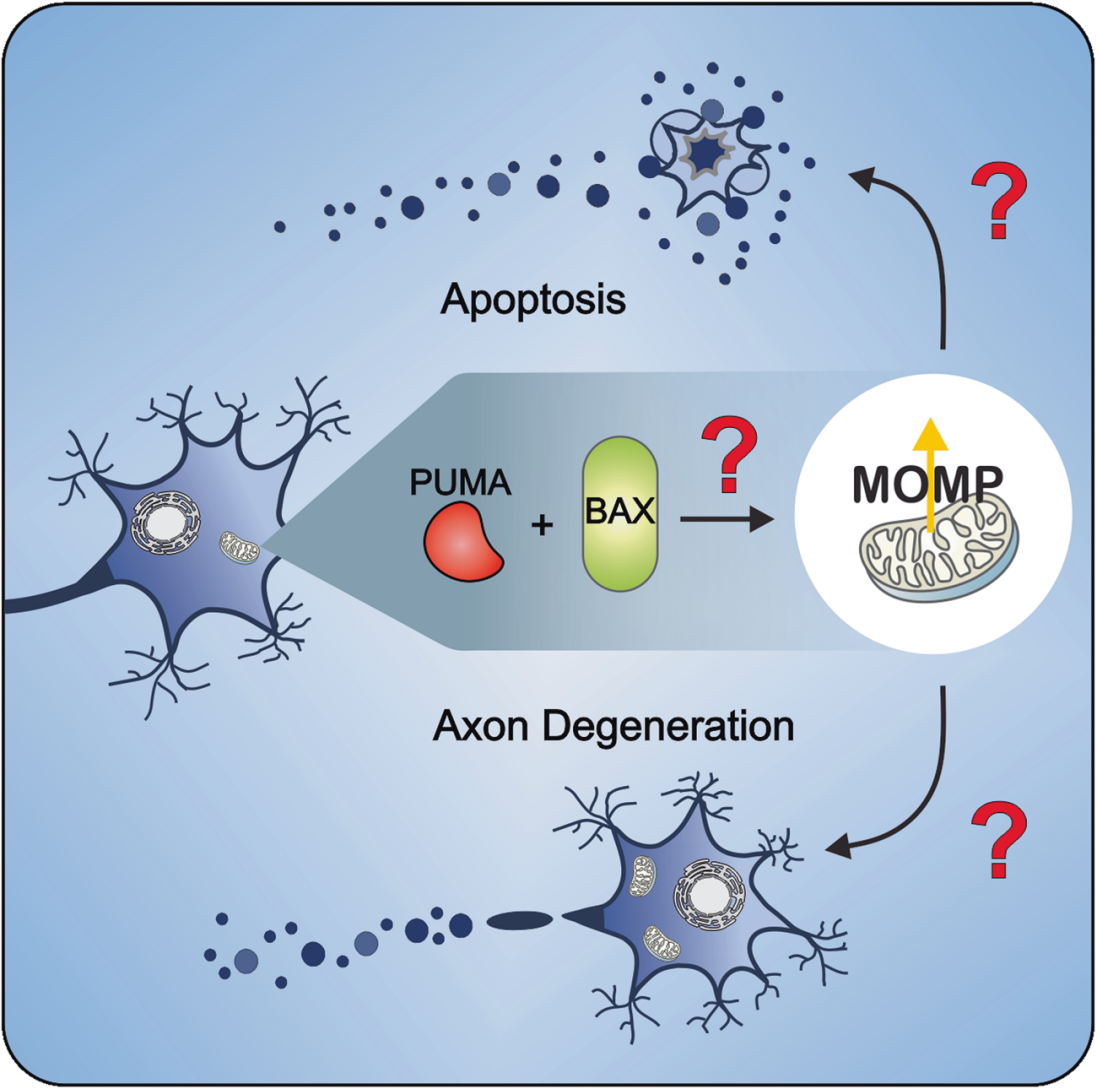
The BCL-2 family proteins PUMA and BAX regulate axon degeneration and apoptosis. This unique reliance on only two proteins affords the possibility of pharmacological inhibition to prevent both process from occurring with minimal effects in other tissues. However, detailed understanding of the mechanism(s) of PUMA and BAX induced MOMP and necroptosis are first required.

Unlike other cells, neurons regulate the degeneration of a large part of the cell, the axon, without the cell dying. Surprisingly, neurons use BCL-2 family proteins to regulate both cell death and axon degeneration. Moreover, despite the redundancy that exits within the BCL-2 family that presumably enables cells to respond differently to many stimuli, several types of neurons require only PUMA and BAX to execute many instances of apoptotic and axon degeneration events (Fig. 4). This unique reliance on only two BCL-2 family proteins affords the possibility of pharmaceutical intervention without serious effects in other tissues where the role of PUMA and BAX is more redundant. To take the advantage of the unique regulation of apoptosis and axon degeneration in neurons, it is important to more fully understand the detailed mechanism(s) of PUMA- and BAX-induced MOMP (Fig. 4). Such information is a key to the development of BAX inhibitors that will ultimately save neurons in a wide variety of neurological conditions with minimal effects on other tissues. In addition to PUMA and BAX, other BCL-2 family proteins have been shown to contribute to the regulation of both apoptosis and other types of neuronal death (necroptosis and ferroptosis) and Wallerian axon degeneration. Thus, a more detailed understanding of these processes will likely reveal other potential therapeutic targets. Importantly the imminent development of specific small-molecule inhibitors of BAX will provide the initial tools needed to parse mechanistic details of the coordinate regulation of apoptosis, necroptosis, and other forms of cell death in the pathology of neurodegenerative disease.
